# Supplementary material for: Azacitidine vs. Decitabine in Unfit Newly Diagnosed Acute Myeloid Leukemia Patients: Results from the PETHEMA Registry
Source: Cancers (Basel). 2022 May 9;14(9):2342. doi: 10.3390/cancers14092342 (PMC9105404; doi:10.3390/cancers14092342)
Supplement: Supplementary file 1 [file cancers-14-02342-s001.zip › cancers-1698139-supplementary.pdf]

cancers

MDPI

Azacitidine vs. Decitabine in Unfit Newly Diagnosed Acute Myeloid Leukemia Patients: Results from the PETHEMA Registry

Jorge Labrador [1,*], David Martínez-Cuadrón [2], Adolfo de la Fuente [3], Rebeca Rodríguez-Veiga [2], Josefina Serrano [4], Mar Tormo [5], Eduardo Rodriguez-Arboli [6], Fernando Ramos [7], Teresa Bernal [8], María López-Pavía [9], Fernanda Trigo [10], María Pilar Martínez-Sánchez [11], Juan-Ignacio Rodríguez-Gutiérrez [12], Carlos Rodríguez-Medina [13], Cristina Gil [14], Daniel García Belmonte [15], Susana Vives [16], María-Ángeles Foncillas [17], Manuel Pérez-Encinas [18], Andrés Novo [19], Isabel Recio [20], Gabriela Rodríguez-Macías [21], Juan Miguel Bergua [22], Víctor Noriega [23], Esperanza Lavilla [24], Alicia Roldán-Pérez [25], Miguel A. Sanz [2], Pau Montesinos [2,*] and (on behalf of PETHEMA group) [†]

Supplementary Figure S1. Univariate analyses of factors influencing response to azacitidine and decitabine.

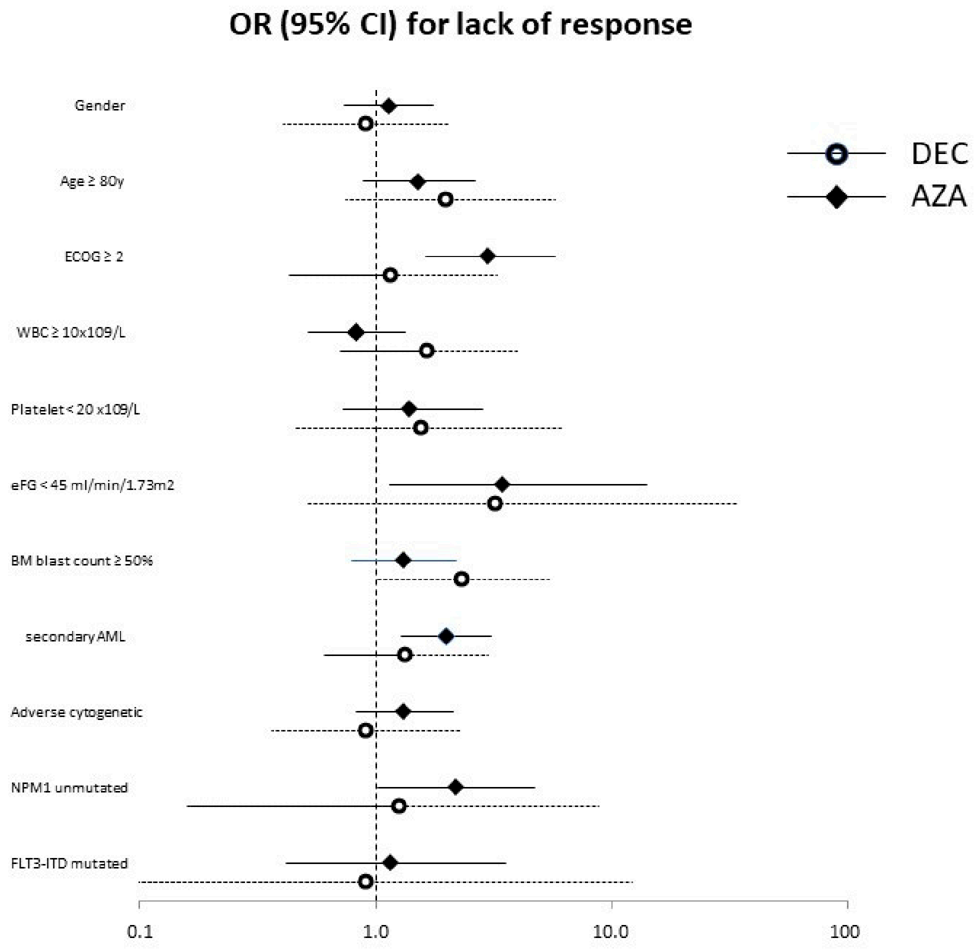

AML, Acute myeloid leukemia; AZA, azacitidine; CR, complete remission; CRi, complete remission with incomplete blood count recovery; DEC, decitabine; ECOG, Eastern Cooperative Oncology Group; eGFR, estimated glomerular fil-trate rate;

FLT3, FMS-like tyrosine kinase 3; ITD: internal tandem duplication; NPM1, Nucleophosmin1; ORR, Overall response rate (CR + CRi + PR); PR, partial remission; WBC, White blood cells.

Supplementary Figure S2. Univariate analyses of factors influencing 120-day mortality after azacitidine and decitabine.

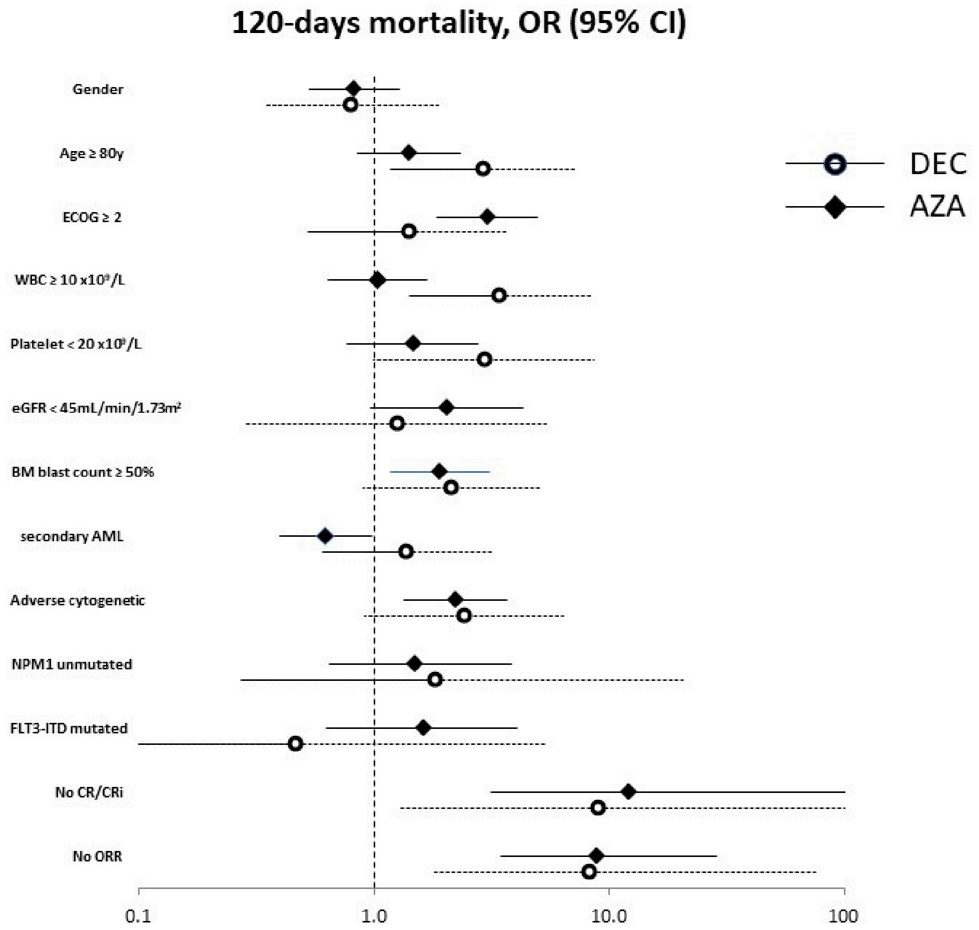

AML, Acute myeloid leukemia; AZA, azacitidine; CR, complete remission; CRi, complete remission with incomplete blood count recovery; DEC, decitabine; ECOG, Eastern Cooperative Oncology Group; eGFR, estimated glomerular filtrate rate; FLT3, FMS-like tyrosine kinase 3; ITD: internal tandem duplication; NPM1, Nucleophosmin1; ORR, Overall response rate (CR + CRi + PR); PR, partial remission; WBC, White blood cells.

Supplementary Figure S3. Event free survival and Relapse free survival for azacitidine vs. decitabine.

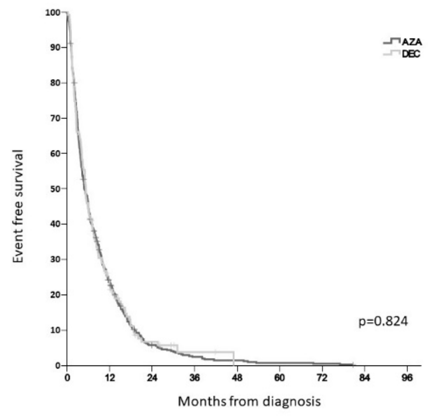

(**a**)

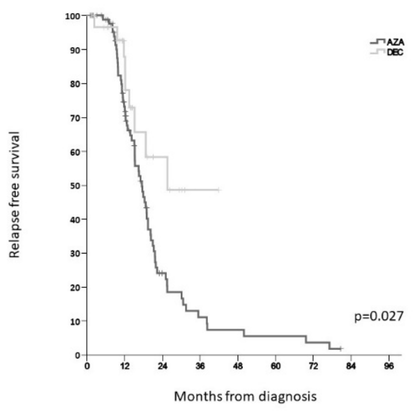

(**b**)

Figure 3. (**a**) Event free survival for azacitidine vs. decitabine; (**b**) Event free survival and Relapse free survival for azacitidine vs. decitabine. AZA, azacitidine; DEC, decitabine.

Group Name: Programa Español para el Tratamiento de las Hemopatías Malignas (PETHEMA)
